# Etiopathogenesis of Differentiated Thyroid Carcinomas

**DOI:** 10.3889/oamjms.2016.086

**Published:** 2016-08-02

**Authors:** Tanja Makazlieva, Olivija Vaskova, Venjamin Majstorov

**Affiliations:** *Institute of Pathophysiology and Nuclear Medicine “Akademik Isak Tadzer”, Medical Faculty, Ss Cyril and Methodius University of Skopje, Skopje, Republic of Macedonia*

**Keywords:** Papillary thyroid carcinoma, Follicular thyroid carcinoma, radiation exposure, MAPK signalling network, PI3K-AKT pathway

## Abstract

**INTRODUCTION:**

Thyroid malignomas are a heterogeneous group of neoplasm consisting of most frequent differentiated encountered carcinomas, papillary and follicular thyroid carcinoma, then medullary thyroid carcinoma originating from neuroendocrine calcitonin-producing C-cells and rare forms of thyroid lymphomas arising from intrathyroidal lymphatic tissue, thyroid sarcomas and poorly differentiated anaplastic thyroid carcinoma. There are increasing numbers of epidemiological studies and publications that have suggested increased incidence rate of thyroid carcinomas. We have read, analysed and compare available reviews and original articles investigating different etiological factors in the development of thyroid carcinomas through Google Scholar and PubMed Database.

**DISCUSSION:**

Aetiology involved in the development of thyroid carcinomas is multifactorial and includes external influences, as well as constitutional predispositions and genetic etiological factors. The actual effect of environmental and constitutional factors is on promoting genetic and epigenetic alterations which result in cell proliferation and oncogenesis. Until now are identified numerous genetic alterations, assumed to have an important role in oncogenesis, with MAPK and PI3K-AKT as crucial signalling networks regulating growth, proliferation, differentiation and cell survival/apoptosis.

**CONCLUSION:**

This new molecular insight could have a crucial impact on diagnosis and also on improving and selecting an appropriate treatment to the patients with thyroid malignancies.

## Introduction

### Classification of thyroid malignomas

Thyroid malignomas (TM) are a heterogeneous group of neoplasm, which according to histopathological features are grouped in neoplasm originating from epithelial follicular cells, the differentiated thyroid carcinomas with the most frequent variants, papillary and follicular carcinoma and medullary thyroid carcinoma originating from neuroendocrine calcitonin-producing C-cells. Very rare forms of primary thyroid neoplasm are thyroid lymphomas arising from intrathyroidal lymphatic tissue and thyroid sarcomas, developing probably from cells of the intrathyroidal connective tissue [1, 2]. In separate category is poorly differentiated anaplastic thyroid carcinoma, which according to prognosis is one of the most aggressive tumours, but is very rare with only 1 – 2 % of all TM [3].

Papillary thyroid carcinoma (PTC) is the most common variant of TM, with the representation of around 80% according to the most studies, but this category of TM isn’t homogenous. There are several histopathological variants of PTC described which are: typical variant, follicular variant, microcarcinoma, tall cell, oncocytic, columnar cell, diffuse sclerosing, solid, clear cell, cribriform morular, macrofollicular, PTC with fasciitis – like stroma, relatively rare and newly described Warthin – like PTC, mixed papillary and medullary carcinoma and papillary with dedifferentiation in anaplastic carcinoma [4-7].

Category of well-differentiated TM includes also follicular carcinoma (FC). According to appropriate chapter of *WHO Classification of Tumours of Endocrine Organs*, FC is defined as malignant epithelial tumours with follicular cell differentiation, without the presence of specific diagnostic nuclear characteristics for PTC. Data from the study of Manuel Sobrinho-Simões et al. indicate declining incidence of this category TM from entirely not known reasons [8]. Frequent detection of follicular variant of PTC and worldwide conducted projects for correction of iodine deficiency are assumed main reasons for detected decline in the incidence rate of FC. According to the epidemiological data, FC is accounting for about 10% of all thyroid carcinomas, but the biggest diagnostic challenge is exact discrimination between follicular adenomas (FA) from minimally invasive follicular thyroid carcinomas and encapsulated follicular variant of PTC [8]. Among other variants of TM, FC includes also Oncocytic Hurthle cell variant [2, 9, 10].

### Epidemiology

Epidemiological data indicate that TM are the most frequent endocrinological neoplasm and according to the latest evaluations they participate with around 1 % in all malignomas [11-13]. An epidemiological study from US National Cancer Institute Surveillance, Epidemiology and End Results (SEER) program for the period 1975 to 2012 year registered 13.5 newly diagnosed cases of thyroid carcinomas per 100 000 people per year. The mortality rate from thyroid carcinomas was 0.5 deaths per 100 000 persons, per year and is estimated that approximately 1.1 % of the population will be diagnosed with TM at some point in life, extrapolated from statistical data for the American population for the period 2011 – 2012 [14]. According to the data published in guidelines of American Thyroid Association (ATA, 2015) incidence rate per year for well-differentiated thyroid carcinomas almost tripled from only 4.9/100 000 in 1975 to 14.3/100 000 in 2009. Analysis showed that almost all increase is due to the rise in the incidence rate of PTC thus 39% of the cases detected in 2008/09 were < 1cm [15]. Carlo La Vecchia et al. using available data from WHO, published detailed epidemiological study regarding incidence, prevalence and mortality rate of TM in countries from Europe, North, Central and South America and Asia. Main conclusions from the study were that the highest increase in mortality rate was detected in countries of Central America and Asia and in countries of East and Central Europe and lowest mortality in countries of Western Europe and North America. Valuable acknowledgement from this study was that there is a continuous decrease in overall mortality from TM, although in the same time increase in incidence rate of thyroid carcinomas in all countries included in epidemiological analysis through all continents was detected. Investigations were in the direction of detecting potential exposure to geographical risk factors responsible for the change in incidence and mortality rate. Some of the regions with the high mortality rate included territories with iodine deficiency in the past, with also highest incidence of benign thyroid disorders, goitre and benign thyroid nodules/adenomas, which are considered as one of the most significant risk factors for the development of TM [16]. Conclusions from this and similar studies suggest that increased incidence of thyroid carcinomas may be also due to the improvement in diagnostic tools and early detection of small microcarcinomas less than 1 cm in diameter. According to others one of the dominant risk factors is increased exposition to radiation, mostly due to inappropriate use in diagnostic and therapeutic purposes [17, 18].

## Discussion

### Environmental etiological factors

Etiological factors involved in the development of TM are multifactorial and include external influences and constitutional predispositions and genetic etiological factors [1]. Because of the heterogeneous character of this neoplasm, according to originating cells, histopathological features, genetic factors, differences in therapeutic protocols and prognosis, the focus of this review will be on the etiopathogenesis of well-differentiated thyroid carcinomas, originated from epithelial follicular cells. The most studied risk factor in the oncogenesis of TM is radiation exposition during childhood, confirmed in numerous publications that significantly increase the risk for development of thyroid carcinomas. Unfortunately first observations about the connection between radiation exposition of neck during childhood and increased risk for development of thyroid carcinomas are studies of children which in period from 1940 till 1950 underwent local irradiation treatment of the head and neck for infection and inflammation of tonsillar and nasopharyngeal region and also irradiation therapy of acne and thymus [19]. Long term follows up of this children, showed the frequent occurrence of nodules in the thyroid gland and an insignificant number of these children was diagnosed thyroid carcinoma [20, 21]. Later gathered data of population from Hiroshima and Nagasaki atomic bombing were in concordance with these first observations. Furukawa et al. published one of the most thorough analyses on this issue. His study is authentic and gives important contribution because of the fact that evaluation and follow-up of a large number of exposed subjects in period 1958 – 2005 were conducted including 105,401 exposed persons. Using linear dose – response model, increased the relative risk of 1.28 (95% confidence interval 0.95-2.7) for thyroid carcinoma after radiation exposition of 1 Gy was calculated at the age of 60 years, after acute exposition in childhood at 10 years age. Results of this study showed that increased relative risk for development of thyroid carcinoma in a population that was exposed to radiation in early childhood; persist even after a long latent period of 50 years, although this relative risk is very low [22, 23]. Facts for the deleterious effect of radiation exposition in the development of thyroid carcinoma in population from Hiroshima and Nagasaki later were confirmed after Chernobyl nuclear power plant accident. Increased incidence rate was detected even in the first 3 – 4 years after the accident, especially in the youngest population in the age group up to the age of 4 years. Highest incidence increment of paediatric thyroid carcinomas was detected in the region of Chernobyl and in place Gomel in Belarus as a result of exposition to ^131^I radioisotope and the additional predisposing factor was iodine deficiency of the population [24]. Schonfeld et al. suggested that latent period between exposition and manifestation of the disease was at least 5 years, while in the study of Cardis et al. incidence increment after irradiation in Chernobyl was detected after only 3 years. The relative risk for development of TM was highest after a period of 20 years, and it reduces after this period. The evaluated relative risk exists at mean exposition dose of 10 cGy and above this exposition dose up to 1500 cGy exists linear dependence between irradiation and risk for development of TM. Higher expositions above 1500 cGy are associated with a reduction in relative risk, probably because of the cytotoxic effect of this high exposition doses. Besides irradiation, important constitutional factor was the age of the children, it was detected that age above 15 years is accompanied with reduced risk for development of TM after exposition to irradiation [19].

Another environmental factor in the etiopathogenesis of TM is assumed to be iodine diet intake (nutritive iodine deficiency or sufficiency) [23]. It is well-known fact that low but also high intake of iodine may result in changes of TSH. Experimental animal studies showed that both conditions could be stimulating cancerogenic factors. Studies with prolonged nutritive iodine deficiency with simultaneous use of known cancerogenic agents such as chemical mutagens resulted in a significantly higher incidence of TM in experimental animal studies. According to this survey iodine deficiency has impact more like promoter, not like direct cancerogenic agent through inducing increase of TSH, and TSH stimulation stimulates autocrine/paracrine regulated thyroid EGF (Epidermal growth factor) stimulated proliferation, lowering in the same time TGFβ1 (Transforming growth factor β1), which acts as a negative factor of thyroid cell proliferation, which results in increased angiogenesis and promoting tumor growth [25]. A number of studies showed a high incidence of follicular and anaplastic carcinomas in population from iodine deficiency regions. Studies with increased iodine ingestion and risk of TM are inconclusive. Rossing et al. in a study conducted among Asian women originating from Philippines and Japan but living in America, detected higher incidence in those born in Asia and later settled in America in comparison to those born in America. Authors concluded that this difference in incidence rate may be the result of environmental influences (perhaps due to nutritive differences), which act in the later stages of cancerogenesis and that those influences are reversible [26].

### Constitutional etiological factors

Pre-existing benign thyroid disease is also one of the risk factors for the development of TM. According to several case-control studies and prospective studies, both benign thyroid nodular/multinodular disease and goitre, autoimmune disorders (Graves’s disease and Hashimoto disease) are described as conditions for increased risk for developing TM, although biases cannot be excluded [27].

Because of significant difference in incidence rate of TM between females and males after puberty and in reproductive period, it was suggested possible oestrogen effects in the development of thyroid carcinomas. Cheng GG et al, in their in vitro study, demonstrated that oestrogen receptors are present on thyroid cancer cell lines [28].

Obesity is another risk factor detected through numerous case-control studies. The exact pathophysiological mechanism is not entirely known, but an increase of TSH and interplay of TSH and insulin – like growth factor 1 inactivation of MAPK and PI3K pathways in obese patients may be enrolled in pathogenesis [29].

High incidence of papillary carcinoma is registered in patients with familial adenomatous polyposis and Cowden’s disease (multiple hamartoma Sy). Thyroid carcinomas are mostly sporadic, but in 5% are with familial appearance, with determined few loci in predisposing genes in this families [30].

### Genetic alterations

Further elucidation of oncogenesis of thyroid carcinomas can be achieved with recent developments in molecular biology and identification of genetic abnormalities in patients with TM. Some identified genetic alterations in patients with thyroid carcinomas are changes in tyrosine kinase domain of RET gene in 15 – 33 %, RAS mutations detected in 10% and B-RAF mutations in 40-60% of cases. Several molecular alterations were identified in last year’s, which included genetic and epigenetic alterations in signalling pathways, like MAPK (Mitogen-activated protein kinases are known also as ERK – extracellular signal-regulated kinases) pathway responsible for tumour initiation and PI3K-AKT signalling pathway for progression and dedifferentiation of thyroid carcinomas. Molecular mechanisms involved in the pathogenesis of TM can be divided into genetic and epigenetic alterations. Genetic alterations can be also classified as nuclear genetic mutations, genetic rearrangements and loss of heterogeneity, while epigenetic changes can be DNA mutilation, histone modification and genetic silencing through microRNA. Nuclear genetic mutations include activation of MAPK pathway. Activation of this pathway in thyroid carcinomas is through RET/PTC rearrangement, RAS mutations and BRAF mutations. MAPK pathway is a network of three kinases which successfully activates one another through sequential phosphorylation in response to various stimulating factors, like growth factors, cytokines, neurotransmitters, cellular stress and cell adhesion. This complex activating network participates in the regulation of numerous cellular processes, among which is control of cell growth, differentiation, cellular adaptation to chemical and physical stress. In RAF-RAS/MEK/ERK pathway signalling transduction starts with activation of small molecule GTPase RAS, through receptor tyrosine kinases, G – protein coupled receptors and/or integrins. These membrane proteins are a group of signalling complexes which activates RAS proteins through changes in RAS – GTP, active or RAS – GDP, inactive form. RAS mutations cause inactivation of GTP are resulting in permanent active RAS- GTP form. There are 3 isoforms of RAS: HRAS, KRAS and NRAS found in thyroid tumours. RAS mutations are usually found in follicular thyroid adenomas, suggesting that those mutations occur early in the development of premalignant lesions and additional genetic alteration can trigger malignant transformation to follicular adenomas. Presumably, KRAS and concomitant PTEN deletion can induce malignant transformation in the aggressive form of follicular thyroid carcinoma. Mutations and deletions of tumour suppressor PTEN gene are genetic alterations which activate PI3K-AKT pathway and are a genetic basis for follicular thyroid carcinoma in Cowden’s Sy [31]. While RAS mutations are characteristic for follicular thyroid neoplasms, RET/PTC proto-oncogenes are almost exclusively found in PTC. Development of TM very similar to PTC in humans was detected in experimental models of mice with RET/PTC expression. There are variations in the prevalence of RET/PTC oncogenes in PTC, but the highest frequency was detected in tumours occurring in children after radiation exposition [32]. At least 10 different RET/PTC oncogenes are described as a result of the fusion of tyrosine kinase domain on RET 5’ part of different genes. RET/PTC1 and RET/PTC3 are most common types of all rearrangement with >90% participation [33]. Another relatively often detected genetic alteration, which is experimentally proven in around 45% of PTC is T1799A transverse point mutation of BRAF (mutation of the gene for B- a type of RAF kinase), manifesting with an expression of BRAF - V600E mutated protein, resulting in successive activation of serine/threonine kinase. One extensive multicentric study showed a strong association of BRAF - V600E mutated protein with the worst prognostic outcome and more aggressive form of PTC, usually associated with tall cell variant of PTC and lost of iodine avidity and, because of that, resistance on radioiodine treatment [34].

Besides changes in MAPK pathway important for oncogenesis are changes in the PI3K-AKT signalling pathway, which stimulates cell proliferation. PTEN tumour suppressor gene (phosphate and tensin homolog), localised on the 10q23 chromosome is an inhibitor of this pathway, acting as a natural restrictor of cell proliferation, preventing the tumour growth [35]. Various changes, like mutations, deletion and silencing of this gene are described. TP53 is tumour suppressor gene localised to chromosome 17 and it is considered that dedifferentiation in the evolution of the tumours may be due to the mutations of this gene. Genetic rearrangement like translocation results in creating a new protein with oncogenic features. W Chien et al in their study *Molecular biology of thyroid cancer*, indicate the presence of chromosomal translocation t(2;3)(q13;p25), PAX8/PPARγ rearrangement, with a fusion of PAX8, thyroid-specific transcription factor to PPARγ, nuclear hormone receptor involved in differentiation of cells. This genetic rearrangement was found in 36% of FC (Follicular thyroid carcinoma), 11% of Follicular adenoma and 13 % of Follicular variant of PTC [36, 37]. Understanding of molecular processes contributed to the thorough investigation of molecular pathogenesis in well-differentiated thyroid carcinomas.

**Figure 1:**
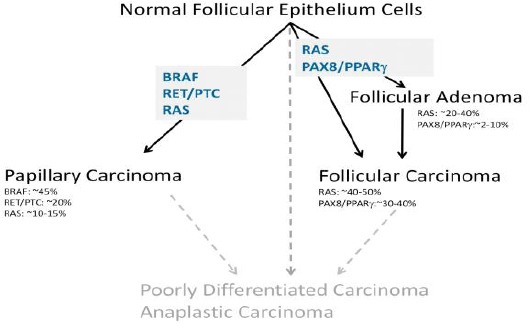
Molecular mechanisms in Thyroid carcinomas. (Asuragen Inc. White Paper: Molecular Pathogenesis of Thyroid Cancer)

In conclusion we can resume that in the development of TM complex mechanisms of interaction between environmental factors, radiation exposition, especially in early childhood, nutritive factors with particular emphasis on iodine intake, chemical mutagen agents and on the other hand internal constitutional predispositions age, sex, obesity and previous thyroid disorders as multinodular goiter could have impact. The actual effect of these environmental and constitutional factors is to promote genetic and epigenetic alterations which result in cell proliferation and oncogenesis. Till now numerous genetic alterations are identified that have an important role in oncogenesis, with MAPK and PI3K-AKT being crucial signalling networks regulating growth, proliferation, differentiation and cell survival/apoptosis. Changes in a different segment of this pathways result in activation of oncogenesis in TM. Different disorders, such as inflammatory, immunological and degenerative processes, could initiate impairment of complex signalling networks and this new molecular insight could have a crucial impact on improving and selecting appropriate treatment for patients with TM [38].
